# An Electroencephalography Bioassay for Preclinical Testing of Analgesic Efficacy

**DOI:** 10.1038/s41598-018-34594-2

**Published:** 2018-11-06

**Authors:** Suguru Koyama, Brian W. LeBlanc, Kelsey A. Smith, Catherine Roach, Joshua Levitt, Muhammad M. Edhi, Mai Michishita, Takayuki Komatsu, Okishi Mashita, Aki Tanikawa, Satoru Yoshikawa, Carl Y. Saab

**Affiliations:** 1 Department of Neurosurgery, Rhode Island Hospital, Providence, RI USA; 2 Department of Neuroscience, Brown University, Providence, RI USA; 3 Laboratory for Pharmacology, Asahi KASEI Pharma Corporation, Shizuoka, Japan; 4 Laboratory for Safety Assessment & ADME, Asahi KASEI Pharma Corporation, Shizuoka, Japan

## Abstract

We present a multimodal method combining quantitative electroencephalography (EEG), behavior and pharmacology for pre-clinical screening of analgesic efficacy *in vivo*. The method consists of an objective and non-invasive approach for realtime assessment of spontaneous nociceptive states based on EEG recordings of theta power over primary somatosensory cortex in awake rats. Three drugs were chosen: (1) pregabalin, a CNS-acting calcium channel inhibitor; (2) EMA 401, a PNS-acting angiotensin II type 2 receptor inhibitor; and (3) minocycline, a CNS-acting glial inhibitor. Optimal doses were determined based on pharmacokinetic studies and/or published data. The effects of these drugs at single or multiple doses were tested on the attenuation of theta power and paw withdrawal latency (PWL) in a rat model of neuropathic pain. We report mostly parallel trends in the reversal of theta power and PWL in response to administration of pregabalin and EMA 401, but not minocycline. We also note divergent trends at non-optimal doses and following prolonged drug administration, suggesting that EEG theta power can be used to detect false positive and false negative outcomes of the withdrawal reflex behavior, and yielding novel insights into the analgesic effects of these drugs on spontaneous nociceptive states in rats.

## Introduction

More than 15% of adults in the United States suffer from chronic pain^1^. Ineffective therapies and narrow therapeutic windows of available drugs^2^ are contributing to the opioid epidemic^3^. The scant success rate of drug development is partly attributed to poor translatability of analgesic efficacy in pre-clinical models, which is based predominantly on behavioral assays. Of these, the most commonly-used are withdrawal reflexes that are qualitative and suffer from a poor temporal resolution. Even when operant behaviors^4^ and other types are considered^5,6^, their relevance to brain states underlying nociception and pain assessment in the clinic is not direct nor at times evident^7^. Hence, there is an emerging interest in novel methods that measure stimulus-independent ‘spontaneous’ pain in an observer-independent manner, especially methods that could be applied to humans. One such approach with a promising translational potential is cortical theta power using quantitative electroencephalography (EEG)^8^.

Earlier studies suggested that resting state EEG oscillations in the theta band are enhanced in neurogenic pain patients and attenuated after therapeutic thalamic lesions^9,10^. Our laboratory was first in reverse-translating these findings to animal models in order to elucidate the mesoscale neural networks underlying pain-induced theta oscillations. In a series of studies, we showed that oscillations within the low frequency theta (4–8 Hz) band are enhanced in various rodent model of pain using spectral analysis of the local field potential^11^, electrocorticography^12^, and EEG^13^. We also demonstrated that pain-induced theta power is reversed upon spontaneous resolution of pain and attenuated following administration of analgesics at effective doses^12,13^. Furthermore, we demonstrated a causal relation between pain-induced theta power, sensory behavior and micro-scale unitary activity in thalamus^14^, and proposed a hypothetical framework of cortical hierarchy mediating pain processing^15^. Other laboratories have since used low frequency oscillations in the context of pain assessment in rodent models^16,17^. For example, Chang *et al*. demonstrated that theta oscillations can be evoked by transient nociceptive stimuli, are developmentally regulated starting at (but not prior to) postnatal day 21, and manifest during adulthood in the paw incision mouse model^16^.

Here, we use EEG theta power to monitor spontaneous nociceptive states in rats and to measure the anti-nociceptive effects of a compound with known analgesic effects in humans (pregabalin), and other drugs with promising (EMA401) or inconclusive results (minocycline) in clinical trials. (1) Pregabalin is a CNS-acting calcium channel inhibitor and first line medication for several chronic pain conditions^2^. (2) EMA 401 is a PNS-acting angiotensin II type 2 receptor (AT2R) antagonist with promising Phase 2 clinical trial results^18^. Several small-molecule AT2R antagonists are known to have anti-nociceptive effects in rodent pain models. To date, only EMA401 has been shown to have analgesic effects in clinical trials after 3 weeks of daily oral administration^18^. EMA300, an analog to EMA401, has been reported to have cumulative efficacy on hypersensitivity induced in a rodent neuropathy model^19^. However, the equivalent optimal dose of EM401 in rats is unknown. (3) Minocycline is a CNS-acting glial inhibitor with mixed results in animal and human studies. According to the literature, the anti-nociceptive effects of 40 mg/kg (i.p.) minocycline manifest upon prophylactic, but not prolonged treatment in the CCI rat model^20,21^. However, prolonged treatment with minocycline (p.o.) at 80 mg/kg has been shown to reverse mechanical allodynia in a rat model of painful diabetic neuropathy^22,23^. Moreover, the analgesic efficacy of minocycline in human studies is inconclusive, even at relatively high doses^24–27^.

Hence, we hypothesized that pain-induced theta power is attenuated only upon administration of a given drug at a therapeutically effective dose. Doses were determined based primarily on published data in equivalent rodent models and administration routes. Otherwise, we performed pharmacokinetic studies and determined sub-therapeutic and supra-therapeutic doses according to cited clinical studies.

We report parallel trends in the reversal of theta power and paw withdrawal latency (PWL) in response to analgesic administration of pregabalin and EMA 401, but not minocycline. We also note divergent trends at non-optimal doses and following prolonged drug administration. Our results highlight the utility of EEG theta power as an interdisciplinary bioassay for the screening of analgesic efficacy in pre-clinical animal models.

## Results

### Experimental design and control experiments

**Figure 1:**
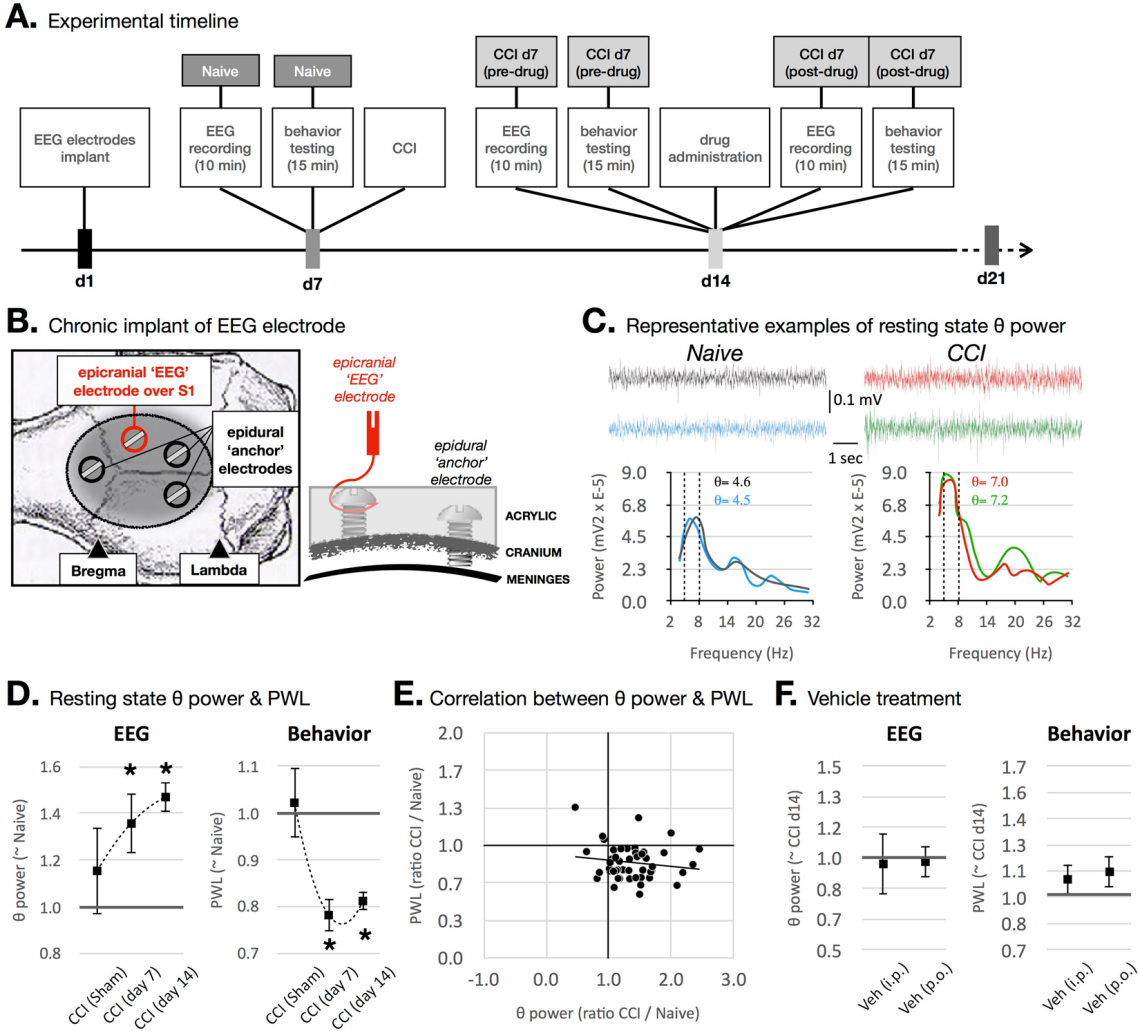
(**A**) Experimental timeline. Rats first undergo implantation of EEG electrodes and allowed one week for post-operative recovery. Second, seven days after EEG implant, EEG recording (~15 min sessions) is followed immediately by behavioral testing (Naive group), then CCI. For testing the analgesic effects of drugs at any given day after CCI (i.e. CCI d7, 11 or 14), EEG and behavioral data are collected prior to, and at several time points after, drug administration. (**B**) EEG implant procedure. (**C**) Representative examples of EEG waveforms from naïve and CCI conditions in the same rat. EEG waveforms were down sampled to 250 Hz and bandpassed between 4 and 8 Hz (theta band). Power spectral density was generated using Fast Fourier transform and mean theta power was computed between 4–8 Hz. (**D**) Time-dependent increase in theta power and decrease in PWL after CCI. (**E**) Correlation between theta power and PWL (*r*^2^ = 0.02, *p* = 0.31). Data are represented as the ratio of CCI d14 relative to Naive. (**F**) Vehicle treatments (i.p., p.o.) had no effect on theta power or PWL 0.5 h after administration. Data are represented as the ratio of vehicle treatment relative to CCI d14.

A total of 67 rats were used. Experimental timeline is shown in Fig. 1A. All rats underwent chronic implant of EEG electrodes with relatively stable recordings throughout the study (Fig. 1B**)**. Figure 1C shows representative EEG waveforms during epochs of awake, resting states under naïve and CCI conditions from the same animal. As we previously reported^12^, rats with CCI showed higher power in the theta range [4–8] Hz compared to naïve. To evaluate these observations quantitively, power spectral densities (PSD) were computed for the time intervals shown, resulting in mean theta power values (Fig. 1C).

All rats receiving CCI developed a significant and time-dependent increase in resting state theta power and a sustained decrease in PWL beginning at day 7 until day 14 after CCI, but not sham CCI surgery (for theta power n = 6 rats in CCI sham, 10 in CCI d7, and 39 in CCI d14; for PWL n = 4 rats in CCI sham, 13 in CCI d7, and 39 in CCI d14; Fig. 1D), suggesting successful reproduction of the neuropathic pain model and confirming our previous findings^11–13^.

Although both theta power and PWL values reliably predict nociceptive states in rats with CCI (i.e. increased theta power and decreased PWL, Fig. 1E, lower right quadrant), no significant correlation between these two measures was found (*r*^2^ = 0.02, *p* = 0.31, n = 67 rats).

In preparation for the drug efficacy experiments, control experiments were performed to rule out confounding variables or adverse effects of animal handling and drug administration. Results showed that neither intraperitoneal (5% Tween 80 in saline, 3 mL/kg) nor oral (0.5% methyl cellulose, 5 mL/kg) vehicle treatment modulated theta power or PWL in rats at day 14 after CCI (n = 5 rats in i.p. group and n = 4 rats in p.o. group; Fig. 1F).

### Pregabalin

The optimal dose for pregabalin was based on a previous study whereby we showed that 10 mg/kg of pregabalin (i.p.) attenuated pain-induced theta power and PWL in rats at day 14 after CCI^13^. To further determine the sub-optimal and supra-optimal doses relative to clinical data, we performed a titration PK study. Pregabalin was administered at 3 and 100 mg/kg (i.p.). Pregabalin plasma concentration has been reported to have good linearity^28^. When comparing our study with a previous report^29^, plasma concentration at 30 min (matching the time point of EEG recording and behavioral testing in this study) showed good linearity at doses of 3 and 100 mg/kg (n = 3 rats per group; Supplemental Fig. 1A). Comparing these rat pregabalin PK data to published human data^30–35^, 10 mg/kg administration in rats [11.3 (4.9) μg/mL] represented as mean or geomean (% coefficient of variation), the same hereafter] corresponded to C_max_ in humans between 300 and 600 mg/day [6.39 (20) and 11.4 (17) μg/mL, respectively]^31^, which are considered to be well within the range of effective analgesic doses in many clinical trials^36^ (Supplemental Fig. 1A and Table 1). A 3 mg/kg dose in rats [3.29 (14) μg/mL] corresponded to the C_max_ value of 150 mg/day in humans [3.18 (21) μg/mL]^31^, a dose with an efficacy considered as insufficient or subtle^35,36^. Rat plasma concentrations at 30 mg/kg [33.0 (5.4) μg/mL] corresponded to slightly higher than steady state level of 600 mg/day in humans (0.244–18.6 μg/mL, represented as range)^33^. On the other hand, 100 mg/kg in rats [99.3 (20) μg/mL] exceeded published reports in human studies. Based on these data, we characterized 3, 10, 30 and 100 mg/kg of pregabalin in rats (i.p.) as sub-therapeutic, therapeutic (i.e. optimal), supra-therapeutic and overdose, respectively, according to clinical standards (Table 1).

**Table 1:** Drug dose in rat relative to therapeutic window in humans.

Mode of action	Efficacy in human	Dose (mg/kg)	Plasma concentration in rat mg/mL (% covariance)		Observation time (min)	Corresponding plasma concentration in human
Pregabalin (CNS, Ca2+ channel)	+(1st line medication)	3	3.29 (14)		30	Sub-therapeutic dose Similar to Cmax of 150 mg/day: 3.18 (21) mg/mL [Chew *et al*.]
		10		11.3 (4.9)*b	30	Therapeutic dose Between Cmax of 300 mg/day: 6.39 (20) mg/mL and 600 mg/day: 11.4 (17) mg/mL [Chew *et al*.]
		30		33.0 (5.4)*b	33	Supra-therapeutic dose Slightly higher than steady state level of 600 mg/day: 0.244–18.6 mg/mL*a [Dworkin *et al*.]
		100	99.3 (20)		30	Overdose
EMA401 (PNS, AT2R)	+(Phase 2)	10	1.59 (70)		30	Therapeutic dose Similar to Cmax of 200 mg/day: 1.01 (65) mg/mL*b [Rice *et al*.]
Minocycline (CNS, glia)	−	80 (p.o.)	12.8 (27)		120	Therapeutic dose (inconclusive) Higher than clinical (200 mg/day) dose: 3.19–4.59 mg/mL*a [Sumitani *et al*.]
			*This study*	*[Lau et al.]*		

Drug plasma concentrations shown as geometric mean (% covariance) except for *a and *b where range and mean.

(% covariance) are presented, respectively.

**Figure 2:**
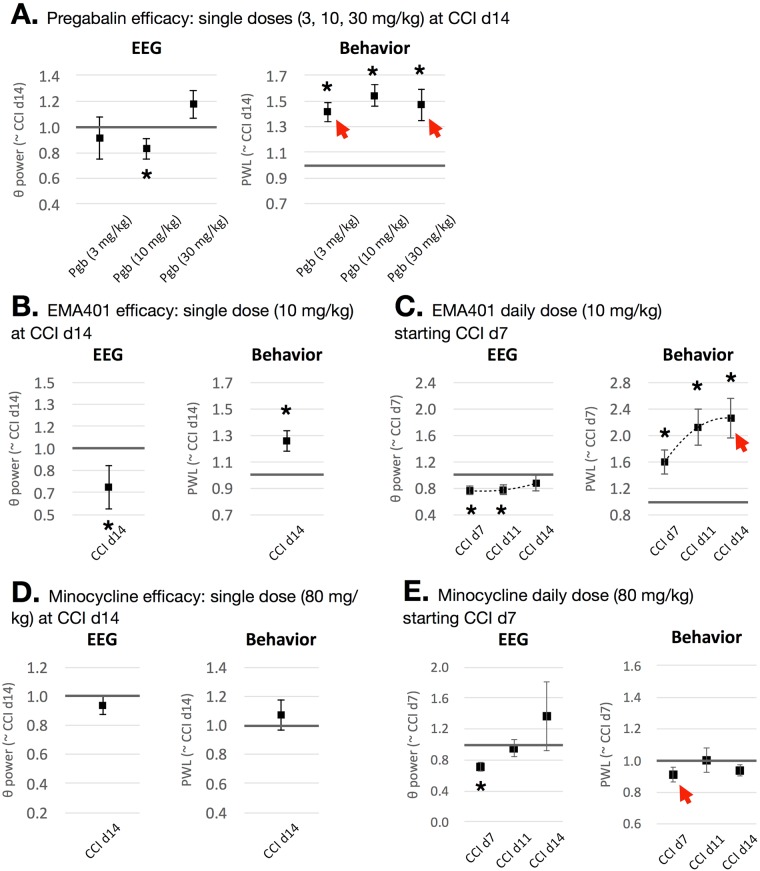
(**A**) The efficacy of pregabalin was analyzed 0.5 h after i.p. administration, whereby 10 mg/kg pregabalin attenuated CCI-induced theta power. Mean theta was represented as the ratio of CCI + pregabalin relative to CCI. Pregabalin reversed PWL at all single-doses tested. Arrows indicate inconsistent results between PWL and theta power (i.e. false positives). (**B**) The efficacy of EMA401 was analyzed 0.5 hr after p.o. administration, which attenuated theta power and increased PWL after single dose administration. (**C**) Daily administration for 7 consecutive days starting at d7 CCI, however, had a short-lasting effect on theta power for the first 5 days but a sustained reversal effect on PWL. Arrow indicates inconsistent result between PWL and theta power (i.e. false positive). (**D**) Single administration of minocycline (80 mg/kg, p.o.) at d14 after CCI had no effects on theta power or PWL. (**E**) The same dose of minocycline (80 mg/kg, p.o.) significantly attenuated theta power at d7 after CCI but not PWL, while having no effects on subsequent days following consecutive daily treatment starting d7 CCI. Arrow indicates inconsistent result between PWL and theta power (i.e. false negative).

We then evaluated the effects of 3, 10, and 30 mg/kg pregabalin (i.p.) on theta power and PWL in rats at d14 after CCI. Pregabalin significantly attenuated theta power in rats with CCI only at the therapeutic dose of 10 mg/kg (0.83 ± 0.12 relative to CCI), but not the sub-therapeutic or supra-therapeutic dose of 3 and 30 mg/kg (0.91 ± 0.11, and 1.24 ± 0.29, respectively; n = 7 rats; Fig. 2A). Interestingly, pregabalin significantly enhanced PWL at all doses (1.42 ± 0.10, 1.54 ± 0.08, and 1.47 ± 0.14 at 3, 10 and 30 mg/kg, respectively, relative to CCI).

### EMA401

EMA401 was administered in rats at 1 and 10 mg/kg (p.o.) and plasma concentration 30 min after 10 mg/kg dose in rats [1.59 (70) μg/mL] was determined to be comparable to clinical data [1.01 (65) μg/mL] (n = 3 rats per group; Supplemental Fig. 1B and Table 1)^18^. We also confirmed that the distribution of EM401 was peripherally restricted (data not shown), as previously reported^37^. In addition, we investigated EMA401’s exposure after daily administration (once/day for 7 days), which was found to be not cumulative. Considering the similar affinity of EMA401 to both human and rat AT2R^38^, we therefore characterized 10 mg/kg (p.o.) as therapeutic dose for EMA401 (Table 1).

We then evaluated the effects of single administration of 10 mg/kg EMA401 (p.o.) on theta power and PWL in rats at d14 after CCI, which resulted in a significant attenuation of theta power (0.71 ± 0.13 relative to CCI) and increase in PWL (1.26 ± 0.11 relative to CCI; n = 6 rats; Fig. 1B). Upon daily administration of 10 mg/kg (p.o.) starting at d7 after CCI, however, the reversal effects on theta power were significant at d7 and d11 but not at d14 after CCI (0.76 ± 0.07, 0.77 ± 0.08, and 0.87 ± 0.12 at day 7, 11 and 14, respectively; n = 6 rats; Fig. 1C), suggesting short-acting effects with regards to resting state nociception, whereas PWL showed significant and cumulative anti-hyperalgesic effects throughout 7 days of treatment (1.60 ± 0.19, 2.13 ± 0.28, and 2.27 ± 0.30 at days 7, 11 and 14, respectively).

### Minocycline

Considering the toxicity of prolonged systemic (i.p.) minocycline relative to the reduced exposure following oral (p.o.) administration, we chose to test the efficacy of single and daily administrations of 80 mg/kg (p.o.) on theta power and PWL. Results show that single administration of minocycline at d14 after CCI had no effects on theta power (0.96 ± 0.13) or PWL (1.08 ± 0.10) relative to CCI (n = 5 rats; Fig. 2D).

Earlier administration of minocycline at d7 after CCI significantly attenuated theta power (0.71 ± 0.06), however, minocycline’s efficacy was short-lasting and non-significant at later time points after daily administration (0.95 ± 0.11 and 1.37 ± 0.45 at day 11 and 14, respectively) (n = 7 rats on d7 and d11 CCI and 5 on d14 CCI; Fig. 2E). With regards to PWL, minocycline had no effect at any day throughout the daily treatment regimen (0.91 ± 0.05, 1.00 ± 0.08 and 0.94 ± 0.04 at day 7, 10 and 14, respectively).

## Discussion

In spite of our increased knowledge of the biology, physiology and neural circuits of pain, little success has been achieved recently in developing new analgesic agents. Pain assessment is a key step in the regulatory process which, regrettably, continues to be based on qualitative criteria reported by the patient (visual analogue scale or VAS) and evoked reflex behaviors in response to acute stimuli in animal models^39,40^. Whereas stimulus-independent behavioral paradigms have been recently proposed (grimace scales^5^, conditioned place preference^41^, burrowing^42^, brain imaging^43^), those tend to be low throughout, subjective and lack the temporal resolution necessary for real-time measurement of spontaneous nociceptive states.

In this study, we present evidence for the positive identification of analgesic efficacy in a rat model of neuropathic pain using a novel EEG theta power assay for the quantitative assessment of spontaneous nociceptive states. We also present data showing the high sensitivity of theta power in detecting false positive and false negative outcomes of the common hyperalgesia test. Three drugs belonging to different families of agents were selected: (1) pregabalin: a CNS target which has a recognized clinical utility as pain therapeutic; (2) EMA401: a PNS target with promising clinical trial results; (3) minocycline: a glial target with inconclusive efficacy (Table 2).

**Table 2:** Summary of analgesic efficacy.

Drug	PREGABALIN			EMA401	MINOCYCLINE	
Target	CNS (Ca + channels)			PNS (AT2R)	CNS (microglia)	
Dose	3 mg/kg (i.p.)	10 mg/kg (i.p.)	30 mg/kg (i.p.)	10 mg/kg (p.o.)	80 mg/kg (p.o.)	80 mg/kg (p.o.)
Pain phase	Late			Late	Late	Early
Equivalent human dose	Low	Optimal	High	Optimal	High	High
Behavioral efficacy (PWL)	+	+	+	+	−	−
EEG efficacy (θ power)	−	+	Side effects	+	−	+
Clinical efficacy (VAS)	−	+	Side effects	+	−	−/+
Different results between PWL & θ power	False positive	True positive	False positive	True positive	True negative	False negative

Single-dose treatment with pregabalin (i.p.) attenuated theta power only when administered at the equivalent therapeutic dose in humans, whereas a sub-therapeutic dose was ineffective and a supra-therapeutic dose resulted in a paradoxical increase in theta power, suggesting potential side effects such as drowsiness which is known to enhance slow wave EEG^44,45^. With regards to behavioral results, pregabalin at all doses reversed PWL to the same level, revealing the ability of theta power to detect putative false positive outcomes based on the PWL test. These results are consistent with clinical data, whereby a sub-therapeutic dose of pregabalin (150 mg/day, corresponding to 3 mg/kg in our study) is effective in reversing allodynia but not spontaneous pain according to the VAS^35^. Pregabalin is known to have anticonvulsant, analgesic and anti-anxiety properties with variable effects on network synchrony over widespread brain regions^46^. Of note, pregabalin has been reported to *enhance* theta power in chronic pain patients^47^. In that study, mean theta power was computed across all EEG electrodes, unlike our focus on S1 in this study. Importantly, the pregabalin dose in the human study was vaguely accounted for (“dose as tolerated”). This is especially concerning since we clearly show here that the effects of pregabalin on EEG are dose-dependent with a tendency for a paradoxical increase in theta at higher doses.

Single-dose treatment with EMA401 (p.o.) reversed both theta power and PWL. Prolonged treatment with EMA401 at the same therapeutic dose, however, had a short-lasting analgesic efficacy measured by EEG and a cumulative effect on withdrawal behavior. In support of our behavioral results, single doses of EMA200, EMA300, and EMA400 (i.p.), which are structurally related to EMA401, have been shown to result in dose-dependent anti-allodynic effects in rats with CCI^38^. It was later reported that EMA300 also confers analgesic properties measured by mechanical allodynia and thigmotactic behaviors in a rat model of antiretroviral toxic neuropathy^19^. Interestingly, the anti-allodynic effects were preserved after prolonged exposure to EMA300 over 3 consecutive days. Our results with regards to prolonged treatment effects on theta power, however, are inconsistent with those of a human study whereby orally administered EMA401 was effective in reducing VAS in patients with postherpetic neuralgia after 3 weeks of treatment, but not before, at a dose of 100 mg/kg twice-daily^18^. This might be attributed to model or species differences, suggesting a previously unknown mechanism of action of EMA401 worth exploring in future studies (see^48^ for a review of EMA401’s developmental program).

Minocycline (p.o.), whose analgesic efficacy is controversial or subtle in clinical studies^24–27^, did not reverse theta power induced during the chronic phase of CCI, but showed subtle attenuation of theta power at an early phase, an effect that was short-lasting upon daily exposure at the same dose. Interestingly, minocycline was reported to be ineffective in ameliorating VAS in patients with neuropathic pain^24^, consistent with the lack of an effect on theta power during the later stage CCI in this study. In support of our behavioral results, daily minocycline treatment (80 mg/kg once/day, p.o.) for 3 weeks started four weeks after induction of diabetes has been shown to attenuate mechanical allodynia while failing to produce significant changes in PWL in a rat model of peripheral diabetic neuropathy^22^. Importantly, it has also been shown that PWL and mechanical allodynia are attenuated when minocycline is administered preemptively prior to CCI (up to 40 mg/kg, i.p.), but not at a later chronic stage^21^. Therefore, theta power is able to detect putative false negative results generated by the PWL test during the early phase of CCI, revealing possible new mechanism of actions for minocycline and glial inhibition in the early phase of neuropathic pain following nerve injury.

In general, the ability to detect false positive or false negative outcomes is crucial to the drug development process. The incorrect assertion of analgesic activity of an agent may arise for several reasons, including the behavioral paradigm in question. To minimize such likelihood, several controls have been recommended, including the concurrent comparisons of a given novel compound with standard, well-characterized agents, consideration of dose-response curves for a side effect profile, detailed pharmacokinetic and pharmacodynamic analysis, and time-dependent effects upon repeated exposure^49^. Our study incorporates most of these recommendations in the experimental design. A few cases in point illustrating the importance of false analgesic activity are the disappointing clinical trial outcomes of presumably promising drug candidates such as CB2 agonist, NK-1 receptor antagonist^50^ and FAAH inhibitor^51^, despite strong evidence for efficacy in pre-clinical studies using behavioral tests, including thermal hyperalgesia^52,53^. Interestingly, thermal hyperalgesia continues to be considered as a primary outcome for the testing of pregabalin’s efficacy in neuropathic pain models including CCI^54,55^.

Although finer temporal resolution at sub-second scales of theta power can be obtained, recording of EEG longer than continuous 30 min was not feasible in our study because EEG recording had to be interrupted to perform behavioral testing. This limitation can be overcome in future studies that do not involve stimulus-dependent behavioral paradigms. Moreover, we do not imply that EEG theta power is the ultimate pre-clinical bioassay. For instance, theta power in this study was measured over S1, which is thought to mediate the detection and localization of pain^56,57^. For other dimensions of the pain experience, for example those that are processed by deep brain structures (amygdala, insula, etc.), EEG would not be a suitable approach and behavioral paradigms such as conditioned place preference or burrowing might be more appropriate. Ultimately, a combination of electrophysiological (EEG), behavioral and possibly fluidic (blood, saliva) biomarker assays might provide a more comprehensive assessment of pain. Because the analgesics in this study were not administered to naive rats, their modulatory effects on normal theta power cannot be ascertained. Overall, waveforms recorded via EEG electrodes placed over S1 cannot be assumed to be generated exclusively from S1. However, we have previously reported that intracortical recordings from S1 layer IV show increased theta power in rats with CCI^11^. Further refinement of recording techniques using high density systems would be needed to more accurately localize the generators of pain-evoked EEG theta.

Our group previously demonstrated that spontaneous states of nociception correlate with increased theta power^11–13^, whereas a transient interruption of theta oscillations corresponds to a time-locked attenuation of hypersensitivity^14^. Furthermore, we showed that theta can be induced in healthy humans subjected to a moderately painful cold-water immersion test^58^. Accordingly, we presented a unifying framework for the hierarchical processing of nociceptive information at the cellular and network levels^15^. Collectively, and in light of evidence presented in this study, we conclude that EEG theta power is a reliable bioassay for testing analgesic efficacy in pre-clinical animal models of pain.

## Methods

### Experimental animals

Adult (200–300 g) male Sprague Dawley rats (Harlan Laboratories, South Easton, MA) were housed under a 12-hour light/dark cycle in a temperature and humidity-controlled environment. Food and water were given ad libitum. All the methods were carried out in accordance with the relevant guidelines and regulations and experiments were approved by the Rhode Island Hospital Institutional Animal Care and Use Committee.

### Electrode implant

These procedures were performed under anesthesia (isoflurane, 2–3.5%). The head was fixed in a stereotaxic apparatus, and skull was exposed after a small skin incision. A stainless steel screw electrode (0–80 ga 1/8 inch, impedance = 0.6 Ω; component Supply Company, Fort Meade, FL) was placed over intact skull (i.e. epicranially) corresponding to primary somatosensory cortex or S1 contralateral to nerve injury (Bregma −2, 2 mm lateral). Minimal craniotomies were used to place 3 stabilization screws (corresponding to Bregma +1.4, 2 mm bilaterally and Bregma −4.8 mm, midline). The EEG screw was threaded with a silver wire and attached to a female miniature pin connector (A&M Systems, Sequim, WA). All screws were anchored to the skull chronically using dental acrylic (Fig. 1B). Signal reference was provided by a silver wire permanently threaded to the skin at the back of the neck.

### Chronic constriction injury (CCI)

The sciatic nerve was exposed unilaterally after skin incision at the mid-thigh level and blunt dissection of the biceps under deep anesthesia (isoflurane, 3%). Four chromic gut ligatures were tied loosely around the nerve 1 mm apart, and the overlying muscles and skin were closed in layers with 4–0 Ethilon sutures. A minor modification was introduced, consisting of loose ligatures, to minimize nerve damage and deafferentation. Rats with this slightly modified CCI procedure gradually develop typical signs of sensory hypersensitivity associated with neuropathic pain, such as guarding the affected hind paw and thermal hypersensitivity. These signs last longer than 2 weeks after CCI, as we previously demonstrated^11–13^. For CCI sham surgery, the sciatic nerve was similarly exposed but not ligated.

### Electrophysiological recording

Pin connectors from EEG electrode was tethered to preamplifier headstage leading to a multichannel amplifier (iso-DAM8A; WPI Inc, Sarasota, FL). EEG waveforms were amplified (DAM80; World Precision Instruments, Sarasota, FL). Then, data were lead to a processing system (micro 1401mkII; Cambridge Electronic Design, CED, Cambridge, United Kingdom) and analyzed offline using Spike 2 (CED). Rats were allowed to move freely during EEG recording in Plexiglas chambers. After 15 minutes of acclimation, EEG recording was performed. Each EEG recording session was 3 to 5 minutes per animal. EEG waveforms were recorded at a sampling frequency of 16.7 kHz, and then down-sampled off-line to 250 Hz. Only data during awake resting periods (defined as alertness but idle with no locomotor behavior) were further analyzed. Potentials generated due to vigorous myogenic activity (such as scratching) were excluded from analysis. These artifacts can be easily identified on the basis of monitoring the animal’s behavior, voltage amplitude, and spectral frequency typically >30 Hz, and they were further corroborated using an automated artifact detection algorithm recently developed in our laboratory (Supplemental Fig. 3)^59^.

### Power spectra density (PSD)

Fast Fourier transform was used to convert EEG waveform from the time domain to the frequency domain. Power values were generated in 27 frequency bins between 3 and 30 Hz. Awake resting periods were selected and Fast Fourier transformed, then average PSD was calculated. Mean PSD theta was computed between 4 and 8 Hz (Fig. 1C). Each ‘period’ was a continuous interval between 7 and 20 seconds, and 3–5 periods were averaged to obtain one value for mean theta per condition.

### Thermal hyperalgesia

EEG recording was performed first for ~10 min during resting states (no stimulation). The rat was then untethered from the EEG leads and placed in the behavioral apparatus for 15 min acclimation, followed by 5 min PWL testing. Thermal sensitivity of the hindpaw was assessed by measuring the latency of the withdrawal reflex in response to a radiant heat source (Paw withdrawal stimulator, Department of Anesthesiology, University of California, SD). A radiant heat source (4.7 A) was applied to the plantar surface of the hind paw. Paw withdrawal latencies (PWL) in response to 4 thermal stimulations were averaged for each paw. Stimulations to each paw were separated by at least 2 minutes intervals.

### Analgesic treatments and drug used

Pregabalin was dissolved in 5% Tween 80 (Sigma Aldrich) in saline formulated for intraperitoneal (i.p.) delivery of 3, 10, and 30 mg/kg in a volume of 3 mL/kg. EMA401 and minocycline (Sigma Aldrich) were suspended in 0.5% methyl cellulose solution (Wako) for oral administration (p.o.) of 10 mg/kg and 80 mg/kg respectively, in a volume of 5 mL/kg.

### Pharmacokinetics (PK) studies

Rats (n = 3 per group) were intraperitoneally treated either with 3 or 100 mg/kg of pregabalin, or orally either with 1 or 10 mg/kg of EMA401, or 80 mg/kg of minocycline. At 0.5, 1, and 2 hours after treatment, approximately 150 µL of blood was collected from the jugular vein using a heparinized syringe with a needle. Blood samples were then centrifuged at 1700 *g* at 4 °C for 10 minutes. Plasma samples were then separated and stored at −20 °C until analysis. Five or 10 μL of plasma samples were diluted with 300 μL of deproteinization solution containing internal standards (5 nM buspirone, 20 nM tolbutamide, 10 nM reserpine in acetonitorile/methanol = 9: 1) and shaken for 5 minutes at room temperature, and centrifuged at 1000 *g* for 15 minutes. Then, the supernatant was analyzed by LC-MS/MS (Xevo TQ-S Tandem Quadrupole Mass Spectrometry, Waters) for quantification of pregabalin. Data analysis were performed with MassLynx Mass Spectometry Software and TargetLynx Application manager (Waters).

### Statistical analysis

Values regarding theta power and PWL are reported as mean ± SEM and then converted to ratio relative to naive or CCI values for standardized representation of the data. One sample t-test was used for comparison between naïve and CCI, and paired t-test was used for comparison between CCI and after treatment in the same animals, respectively. Values regarding drug plasma concentration are reported as geomean (% of covariance) or range. Correlation analysis was used to compare EEG with PWL values. A *P* value < 0.05 was considered statistically significant and denoted by asterisk (*) in figures.

## Electronic supplementary material

Supplementary Information

## Data Availability

The datasets generated during and/or analyzed during the current study are available from the corresponding author on reasonable request.
