# Supplementary material for: An Electroencephalography Bioassay for Preclinical Testing of Analgesic Efficacy
Source: Sci Rep. 2018 Nov 6;8:16402. doi: 10.1038/s41598-018-34594-2 (PMC6219560; doi:10.1038/s41598-018-34594-2)
Supplement: Supplementary file 1 — Supplementary Information [file 41598_2018_34594_MOESM1_ESM.docx]

Supplemental information

**Title: An Electroencephalography Bioassay for Preclinical Testing of Analgesic Efficacy**

**Authors**

Suguru Koyama^a,b,c^, Brian W. LeBlanc^a,b^, Kelsey A. Smith^a,b^, Catherine Roach^a,b^, Joshua Levitt^a,b^, Muhammad M. Edhi^a,b^, Mai Michishita^c^, Takayuki Komatsu^c^, Okishi Mashita^d^, Aki Tanikawa^d^, Satoru Yoshikawa^c^, Carl Y. Saab^a,b*^,

**Affiliations**

^a^Department of Neurosurgery, Rhode Island Hospital, Providence, RI, USA

^b^Department of Neuroscience, Brown University, Providence, RI, USA

^c^Laboratory for Pharmacology, Asahi KASEI Pharma Corporation, Shizuoka, Japan

^d^Laboratory for Safety Assessment & ADME, Asahi KASEI Pharma Corporation, Shizuoka, Japan

* Author to whom correspondence should be addressed

Address: Department of Neurosurgery, Department of Neuroscience, Brown University, 593 Eddy St. Aldrich 402, Providence, RI 02903, USA, Email address: carl_saab@brown.edu

Supplemental Figure 1. A_Time course of pregabalin plasma concentration following
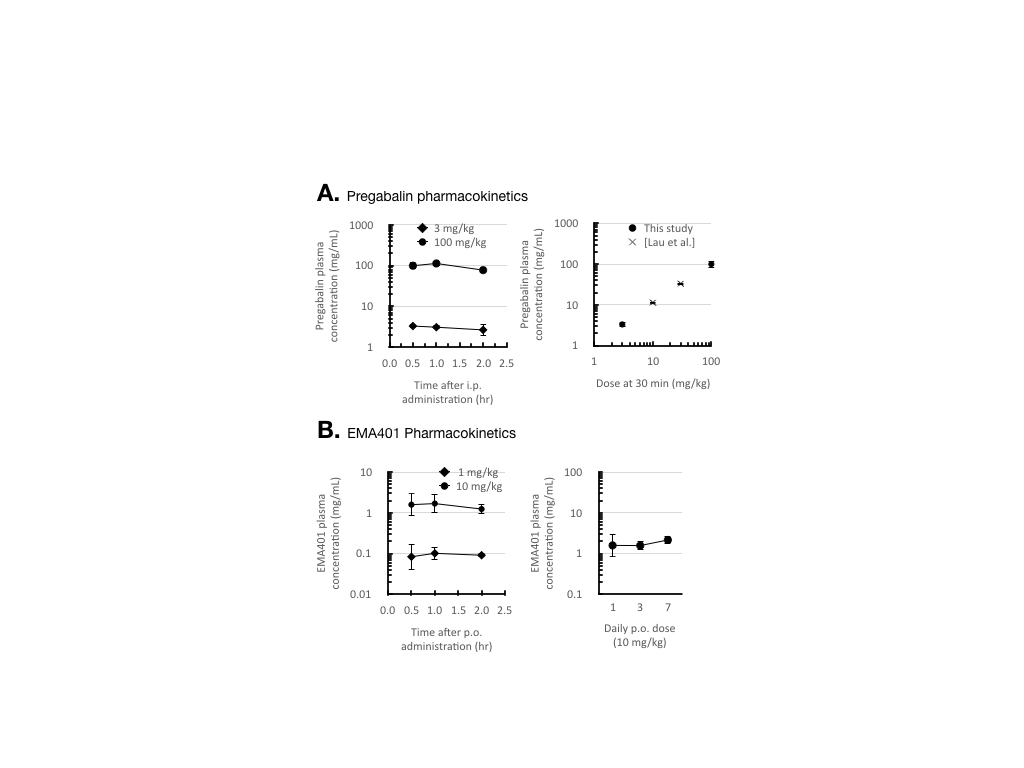
 i.p. administration (3 or 100 mg/kg). Plasma samples were collected at 0.5, 1, and 2 hr. Pregabalin in plasma was quantified by LC-MS/MS analysis. Comparison of pregabalin plasma concentration at 0.5 hr after i.p. administration between our study (3 and 100 mg/kg) and another (10 and 30 mg/kg) (Lau et al. 2013). Note that both the route of drug administration and the vehicle were the same in these two studies. Pregabalin plasma concentration showed good linearity between 3 and 100 mg/kg. B_Time course of EMA401 plasma concentration following p.o. administration (1 or 10 mg/kg). Plasma samples were collected at 0.5, 1, and 2 hr. EMA401 in plasma was quantified by LC-MS/MS analysis. Right panel also shows EMA401 Plasma concentration 2 hr after daily p.o. administration for 7 days.

Supplemental Figure 2. Longitudinal effects of single dose EMA401 (10 mg/kg) on
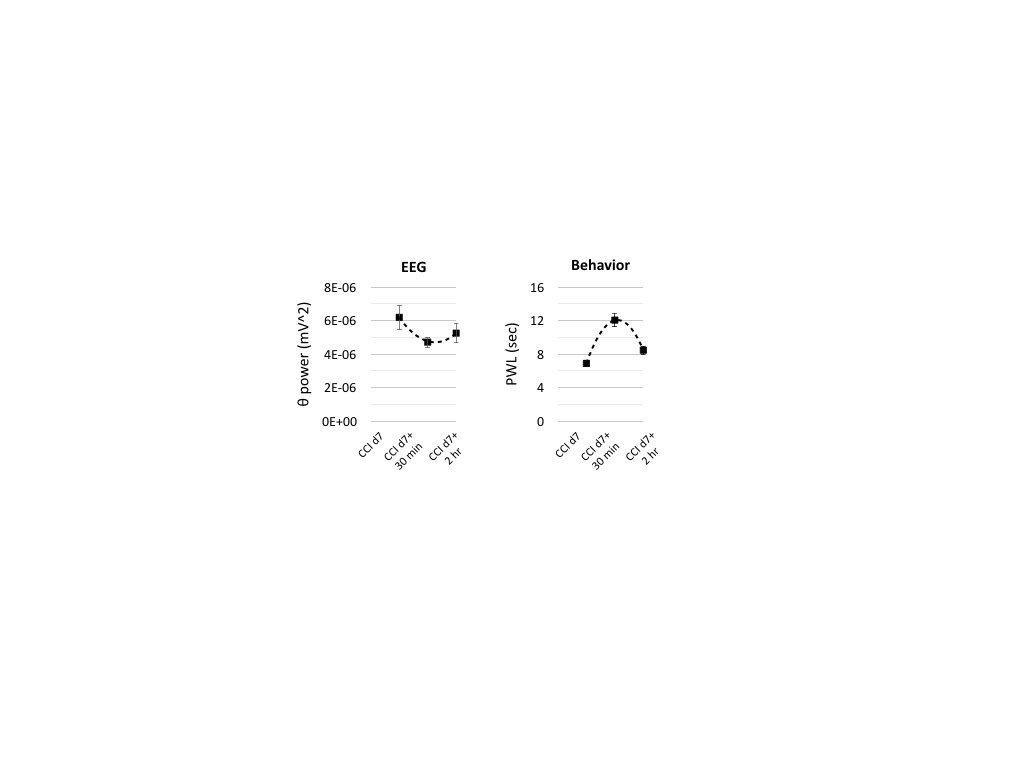
 theta power and PWL in rats with CCI d7 (n=4), showing analgesic effects at 30 min after administration and a tendency for reversal 2 hours after administration, suggesting that variations in theta power are parallel to changes in stimulus-independent nociceptive states during pain, analgesia and reversal of analgesia.

Supplemental Figure 3. Identification of EEG artifacts in awake behaving rats.
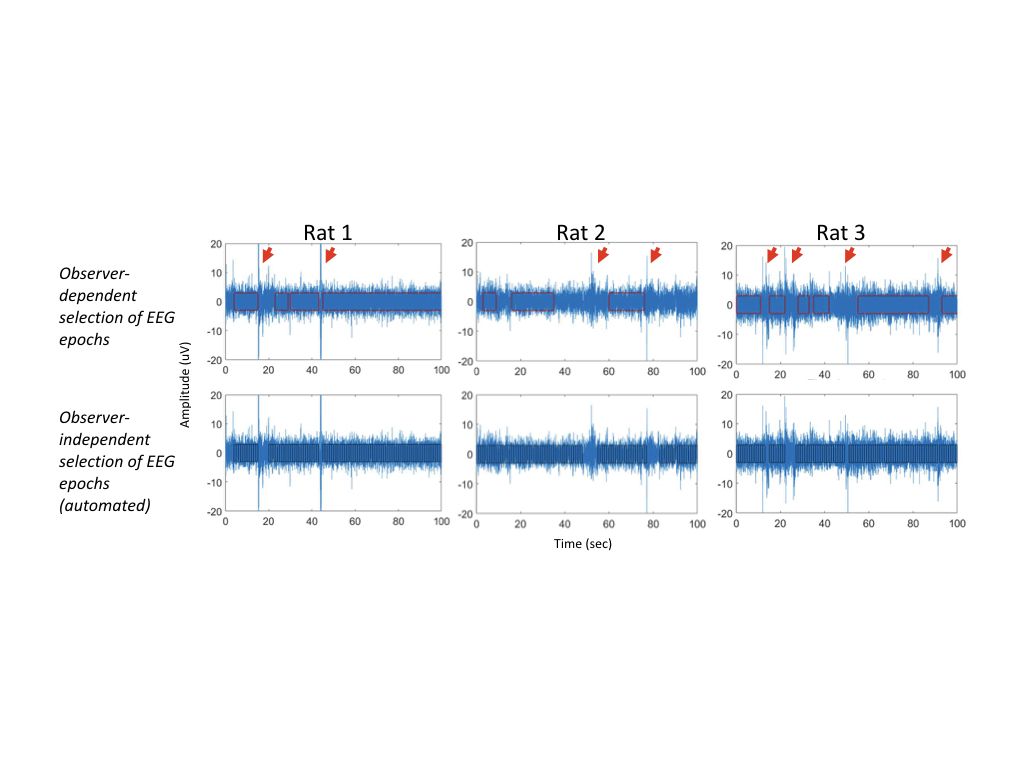
 Observer-dependent selection of EEG epochs was further corroborated by an automated selection method using machine learning. Upper panels represent 100-s long EEG waveforms from 3 randomly-chosen rats with red rectangles showing artifact-free epochs selected by an experimenter (arrows indicate annotated artifacts). Lower panels show same waveforms with artifact-free epochs (black rectangles) detected automatically by a support vector machines (SVM) algorithm developed and validated by our laboratory (window size = 1 sec; see Levitt et al. 2018). Mean theta power computed from artifact-free epochs in upper panels (5.4E-06 ± 5.5E-07) is comparable in amplitude to that in lower panels (4.9E-06 ± 2.8E-07). These representative examples suggest that the selections made by an observer (visual assessment) conform to those made by the SVM (empirical assessment) and that the observer in this case was in fact conservative in the selection compared to SVM.
